# Impact of Coronary Microvascular Dysfunction on Left Ventricular Function After Percutaneous Coronary Intervention: Assessment With Combined Dipyridamole‐Exercise Stress and Myocardial Strain/Work

**DOI:** 10.1111/echo.70419

**Published:** 2026-03-18

**Authors:** Wanyu Zhao, Xiaoli Dong, Xiangyu Ji, Jiong Tang, Lin Ding, Yunfei Zhou, Shuanglan Yu, Jian Li, Haibo Li, Chunfang Yang, Qiuzhe Guo, Zhiling Luo, Yan Shen

**Affiliations:** ^1^ Department of Ultrasound Fuwai Yunnan Hospital, Chinese Academy of Medical Sciences/Affiliated Cardiovascular Hospital of Kunming Medical University Kunming China; ^2^ Department of Cardiology Fuwai Yunnan Hospital, Chinese Academy of Medical Sciences/Affiliated Cardiovascular Hospital of Kunming Medical University Kunming China; ^3^ Department of Interventional Cardiology Fuwai Yunnan Hospital, Chinese Academy of Medical Sciences/Affiliated Cardiovascular Hospital of Kunming Medical University Kunming China

**Keywords:** coronary flow velocity reserve, coronary microvascular dysfunction, dipyridamole‐exercise stress echocardiography, myocardial strain, myocardial work, percutaneous coronary intervention

## Abstract

**Background:**

Coronary microvascular dysfunction (CMD) persists after percutaneous coronary intervention (PCI) in individuals with coronary artery disease (CAD). This study assessed CMD using combined dipyridamole‐exercise stress echocardiography (DExE) and myocardial strain/work to evaluate the impact on left ventricular (LV) function.

**Methods:**

This prospective study enrolled CAD individuals who underwent left anterior descending artery PCI and DExE assessment at 3 months post‐PCI to evaluate myocardial strain/work parameters at rest and stress. The primary endpoint was adverse clinical events, and the secondary endpoint was Seattle Angina Questionnaire (SAQ) health status at 1‐year.

**Results:**

The cohort comprised 84 individuals (54 ± 10 years, 17.9% female). Participants were stratified into two groups by coronary flow velocity reserve (CFVR): CFVR ≥ 2.5 group (*n* = 63) and CFVR < 2.5 group (*n* = 21). Compared with the CFVR ≥ 2.5 group, the CFVR < 2.5 group showed impaired LV functional reserve, with lower global longitudinal strain (GLS), global work index (GWI), and global work efficiency (GWE) at peak exercise and recovery, lower peak exercise global constructive work (GCW), higher recovery global wasted work (GWW), and smaller absolute changes ΔE—R (difference before vs. after exercise stress) in GLS, GWI, and GCW (all *p* < 0.05). In multivariable analysis, peak exercise GCW, recovery GWW, and ΔGCW (E − R) were independent predictors of CMD. At 1 year, no adverse clinical events occurred, and the CFVR ≥ 2.5 group had higher SAQ physical limitation (92.86 ± 11.38 vs. 82.14 ± 17.93), quality of life (95.63 ± 10.57 vs. 83.33 ± 19.90), and summary score (94.05 ± 8.51 vs. 85.71 ± 15.71) than the CFVR < 2.5 group (all *p* < 0.05).

**Conclusions:**

Combined DExE with myocardial strain/work analysis effectively evaluates LV function in CMD. Decreased CFVR post‐PCI is associated with impaired LV function and worse 1‐year quality of life outcomes, underscoring the need for close cardiac function monitoring in post‐PCI CMD individuals.

**Trial Registration:**

URL: https://www.chictr.org.cn/; Unique identifier: ChiCTR2500103229.

## Graphical abstract

Decreased coronary flow velocity reserve (CFVR) was correlated with left ventricular (LV) dysfunction after percutaneous coronary intervention (PCI), as assessed via dipyridamole‐exercise stress echocardiography (DExE). This dysfunction was characterized by lower myocardial strain/work index and worse 1‐year Seattle Angina Questionnaire (SAQ) scores.

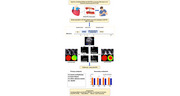

**Abbreviations**
2D‐STE: Two‐dimensional speckle tracking echocardiography
ACS: Acute coronary syndrome;
ACS‐UA/NSTEMI: Acute coronary syndrome‐unstable angina/non‐ST‐segment‐elevation myocardial infarction
AUC: Area under the curve
AVC: Aortic valve closure
CAD: Coronary artery disease
CCS: Chronic coronary syndromes
CFVR: Coronary flow velocity reserve
CMD: Coronary microvascular dysfunction
DExE: Dipyridamole‐exercise stress echocardiography
EAE: European Association of Echocardiography
ECG: Electrocardiogram
GCW: Global constructive work
GLS: Global longitudinal strain
GWE: Global work efficiency
GWI: Global work index
GWW: Global wasted work
HR: Heart rate
IMR: Index of microcirculatory resistance
LAD: Left anterior descending artery
LV: Left ventricular
LVEDVi: Left ventricular end‐diastolic volume index
LVEF: Left ventricular ejection fraction
MI: Myocardial infarction
MW: Myocardial work
NSTEMI: Non‐ST‐segment‐elevation myocardial infarction
PCI: Percutaneous coronary intervention
ROC: Receiver operating characteristic
SAQ: Seattle Angina Questionnaire
STEMI: ST‐segment elevation myocardial infarction
THR: Target heart rate
TIMI: Thrombolysis in myocardial infarction

## 1 Introduction

Percutaneous coronary intervention (PCI) has become a first‐line treatment for various coronary artery disorders [1, 2]. However, even after successful PCI with TIMI III flow, 20%–40% of individuals experienced persistent or recurrent angina, which impaired their quality of life [3, 4], with 50% of these issues due to coronary microvascular dysfunction (CMD) [5, 6].

Dipyridamole stress testing utilizes the coronary steal phenomenon, induced by coronary artery dilation, to diagnose suspected CMD [7]. Guidelines recommend coronary flow velocity reserve (CFVR) measurement for microvascular function assessment [8]. Exercise stress testing activates the sympathetic nervous system, elevating myocardial oxygen consumption via increased heart rate and contractility with concurrent myocardial ischemia [9]. Dipyridamole‐exercise stress echocardiography (DExE) enhances susceptibility to exercise‐induced ischemia by lowering the ischemic threshold, thereby unmasking mild or latent ischemic lesions [10, 11, 12, 13]. DExE, first proposed by Picano in 1988, combines dipyridamole infusion with exercise stress [10], Subsequent studies have demonstrated that this approach improves the diagnostic accuracy for coronary artery disease (CAD) with a favorable safety profile [11, 12, 13]. In addition, two‐dimensional speckle tracking echocardiography (2D‐STE)‐derived myocardial strain and myocardial work are emerging technologies for cardiac function evaluation which demonstrate superior sensitivity for detecting subclinical cardiac dysfunction, even with normal left ventricular ejection fraction (LVEF) [14, 15]. Strain quantifies myocardial deformation using the contraction and relaxation phases [14], while work integrates LV strain with non‐invasively estimated LV pressure, providing an afterload‐adjusted assessment of myocardial performance [15]. However, whether CMD contributes to impaired cardiac function remains unclear.

Herein, we investigated if combined DExE and myocardial strain/work could provide comprehensive cardiovascular assessment by evaluating its impact on left ventricular (LV) function and myocardial contraction reserve in post‐PCI individuals with CAD with LVEF ≥ 50% and persistent CMD.

## 2 Materials and Methods

### 2.1 Study Population

This prospective study included 84 individuals with LVEF ≥ 50% and a diagnosis of chronic coronary syndrome (CCS) or acute coronary syndrome (ACS) unstable angina/non‐ST‐segment‐elevation myocardial infarction (ACS–UA/NSTEM) who underwent PCI for left anterior descending artery (LAD) recanalization between October 2023 and September 2024. CCS and ACS were defined according to contemporary international guidelines [16, 17]. All individuals underwent DExE at Fuwai Yunnan Hospital 3 months after PCI. The study inclusion criteria were: (1) history of PCI with recanalization of the LAD after 3 months; (2) coronary angiography demonstrating successful stent recanalization with TIMI III; and (3) adequate echocardiographic image quality, including clear visualization of LAD coronary flow at rest and apical 2‐, 3‐, and 4‐chamber views acquired at frame rates > 50/s for strain analysis. The exclusion criteria were: (1) significant stenosis (> 70%) in non‐LAD vessels or stent restenosis; (2) baseline wall motion abnormalities or LVEF < 50%; (3) the presence of severe arrhythmias, valvular disease, intracardiac shunt, or cardiomyopathy; (4) failure to achieve ≥ 85% of the target heart rate (THR) during exercise; and (5) suboptimal image quality. All individuals were requested to discontinue medications that may affect the test results 24 h prior to assessment. CMD was defined as a CFVR < 2.5, according to the International Standardization of Diagnostic Criteria for Microvascular Angina issued by the Coronary Vasomotion Disorders International Study Group [18]. The participants were stratified by CFVR into two groups. The study was conducted in accordance with the Declaration of Helsinki and was approved by the Fuwai Yunnan Hospital Ethics Committee (ID: 2024‐013‐01). Informed consent was obtained from all individuals.

### 2.2 Dipyridamole‐Exercise Stress Protocol

**FIGURE 1:**
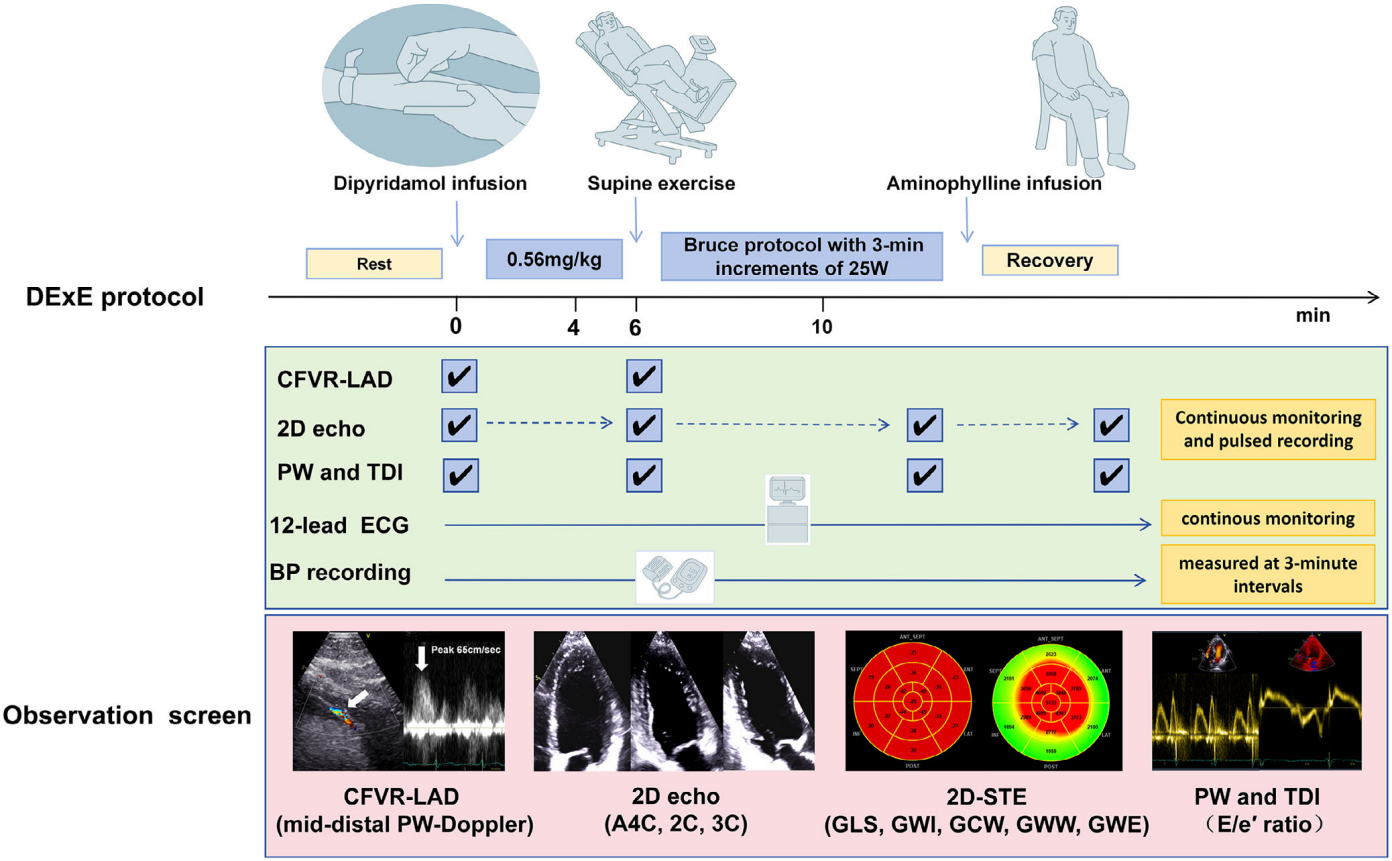
Dipyridamole‐exercise stress echocardiographic protocol for the combined assessment of doppler CFVR‐LAD and myocardial strain/work. DExE, Dipyridamole‐exercise stress echocardiography; CFVR, coronary flow velocity reserve; LAD, left anterior descending artery; 2D echo, two‐dimensional echocardiography; PW, pulsed wave doppler; TDI, tissue doppler imaging; ECG, electrocardiogram; A4C, 2C, 3C, apical 2‐, 3‐, and 4‐chamber; 2D‐STE, two‐dimensional speckle tracking echocardiography; GLS, global longitudinal strain; GWI, global work index; GCW, global constructive work; GWW, global wasted work, GWE, global work efficiency; E/e′ratio, early diastolic mitral inflow velocity to annular velocity.

Examinations were performed using an ultrasound system (Vivid E95, GE Healthcare, Horten, Norway). According to the European Association of Echocardiography (EAE) guidelines [19], the stress protocol started with dipyridamole (140 µg/kg/min i.v. for 6 min). In individuals with normal regional wall motion after dipyridamole infusion, the protocol was achieved by adding supine bicycle exercise at 3‐min increments at a workload of 25 Watts (W) using the Bruce protocol. The test was terminated when 85% of the THR (220‐age) was reached. The exercise phase was followed by a 3‐min recovery period at 25 W, after which aminophylline 120 mg was administered to all individuals. This integrated protocol unites the pharmacological effects of dipyridamole with the physiological responses induced by recumbent bicycle exercise. Figure 1 shows the study flowchart. Continuous electrocardiogram (ECG) recording was maintained throughout the process, and blood pressure was measured at 3‐min intervals. Standard ultrasound apical 2‐, 3‐, and 4‐chamber views were acquired at 50–80 frames/s at baseline, upon completion of dipyridamole infusion, at peak exercise intensity, and during the recovery phase. The coronary velocity of the LAD was measured at rest and at the end of dipyridamole infusion. Peak diastolic coronary flow velocity in the mid‐distal LAD was measured at the peak state, and CFVR was calculated as the ratio of hyperemic to resting velocities (mean of three cardiac cycles).

The diagnostic endpoints of stress echocardiography were: new wall motion abnormalities, ischemic ECG changes (ST deviation ≥ 1 mm), severe symptoms (angina, dyspnea), hemodynamic instability (BP > 220/120 mmHg, drop > 30 mmHg, arrhythmias). Throughout the examination, subject symptoms were closely monitored, and adverse events were carefully observed.

### 2.3 Image Analysis

Echocardiographic data were assessed offline using specialized software (EchoPAC version 204, GE Healthcare). Echocardiographic parameters were measured included left ventricular end‐diastolic volume index (LVEDVi), LVEF (Simpson's biplane method), and early diastolic mitral inflow velocity to annular velocity (E/e′ratio) [20]. Speckle‐tracking analysis was performed using 2D‐strain, with endocardial borders manually traced in apical 2‐, 3‐, and 4‐chamber views and dynamic tracking verification performed, including manual adjustment of poorly tracked segments [21]. The aortic valve closure (AVC) time was identified post‐tracking, and the system automatically generated LV global/17‐segment strain parameters. Myocardial work indices were derived after inputting brachial cuff blood pressure and yielded the following data: (1) Global work index (GWI) representing the total work calculated from mitral valve closure to mitral valve opening in the pressure‐strain loop of the left ventricle; (2) global constructive work (GCW) being the positive work of the aortic valve during contraction (longitudinal shortening) and the diastolic work after closure (longitudinal lengthening); (3) global wasted work (GWW) corresponding to negative work that does not promote ejection or relaxation (elongation of contractile muscle cells, shortening of equal‐volume relaxation muscle cells); and (4) global work efficiency (GWE) that was calculated as GCW/(GCW + GWW) [22].

Two‐dimensional‐STE indices were measured before dipyridamole stress and at maximum dipyridamole stress and maximum exercise stress, where pre‐dipyridamole stress was defined as rest and post‐dipyridamole stress and post‐exercise stress were defined as peak. The difference between dipyridamole stress and before and after rest was calculated as Δ (D − R), and the difference between exercise stress and before and after rest was calculated as Δ (E − R).

### 2.4 Follow‐Up

Clinical outcomes were evaluated at 1‐year follow‐up post‐PCI through scheduled outpatient visits and structured telephone interviews. The primary endpoint was the occurrence of prespecified adverse clinical events within 1 year, including cardiac death, myocardial infarction, coronary revascularization, stroke, heart failure, and new‐onset atrial fibrillation [23]. The secondary endpoint was 1‐year health status assessed using the Seattle Angina Questionnaire (SAQ) [24].

### 2.5 Intra‐ and Inter‐Observer Variability

Fifteen individuals were randomly selected, and two observers, who were blinded to the clinical data, measured myocardial strain and work parameters. The same observers repeated the measurements 1 month later to assess the intra‐ and inter‐observer variability.

### 2.6 Statistical Analysis

Continuous variables are expressed as the mean ± standard deviation (SD) or median (interquartile range [IQR]). The normality of data distributions was examined using the Shapiro–Wilk test, with between‐group analysis using an independent Student's t‐test or the Mann–Whitney U test, as appropriate. Categorical variables were summarized as frequencies or percentages, and comparisons were made using Pearson's chi‐square test. Repeated measures of analysis of variance (ANOVA) with post‐hoc Bonferroni test was used to assess the effectiveness of CFVR over stress phases. For variables with a non‐normal distribution, the Friedman M test was used to compare the GWW and GWE within groups, and the Wilcoxon test was used to assess the differences between the groups. Univariate logistic regression analysis was used to screen for CMD‐related factors. Multivariate logistic regression analysis was based on the results of univariate analysis, and meaningful variables were selected to construct a multivariate model. The receiver operating characteristic (ROC) curve was used to evaluate the diagnostic performance of 2D‐STE and myocardial work (MW) indices in detecting CMD, and the area under the curve (AUC) was quantified to assess predictive accuracy. Intra‐ and inter‐observer agreements were assessed using intraclass correlation coefficients and coefficients of variation. Statistical analysis was performed using the SPSS 27.0 package (IBM, Armonk, NY, USA). A *p*‐value < 0.05 was considered statistically significant.

## 3 Results

### 3.1 Baseline Characteristics of the Study Cohort

Eighty‐four individuals were enrolled in the study, with an average age of 54 ± 10 years and 15 (17.9%) females. Individuals were divided into the CFVR ≥ 2.5 (*n* = 63) and CFVR < 2.5 (*n* = 21) groups. Demographics, biometrics, and indications for PCI are summarized in Table 1. The two groups showed no significant differences in age, sex, BMI, comorbidities, drug use, and laboratory results (all *p* > 0.05). No significant difference was found between the two groups in the proportions of chronic stable angina, ACS–UA/NSTEMI, previous PCI, and previous myocardial infarction (MI) (*p* > 0.05). Regarding the parameters related to coronary artery blood flow, the TMIM frame count in the CFVR < 2.5 group was significantly higher than that in the CFVR ≥ 2.5 group (*p* = 0.044), and the CFVR value of the CFVR < 2.5 group was significantly lower than that of the CFVR ≥ 2.5 group (*p* < 0.001).

**TABLE 1:** Baseline clinical characteristics stratified by CFVR post‐PCI.

Parameter	CFVR ≥ 2.5 group (*n* = 63)	CFVR < 2.5 group (*n* = 21)	*p‐*value
Age, y	56 ± 7	59 ± 9	0.098
Sex, Female	12 (19.1)	3 (14.3)	0.869
BMI, kg/m^2^	25.85 ± 3.28	25.73 ± 2.37	0.878
**Comorbidities**			
Hypertension	35 (55.6)	15 (71.4)	0.199
Dyslipidemia	56 (88.9)	18 (85.7)	0.697
Diabetes	16 (25.4)	7 (33.3)	0.480
Smoking	43 (68.3)	15 (71.4)	0.785
Drinking	24 (38.1)	10 (47.6)	0.441
Atrial fibrillation	1 (1.6)	1 (4.8)	0.440
**Medications**			
Any antiplatelet	13 (20.63)	5 (23.81)	0.759
DAPT	50 (79.4)	16 (76.2)	0.759
Statin	63 (100.0)	21 (100.0)	1.000
β‐blocker	46 (73.0)	15 (71.4)	0.888
ACE‐inhibitor/ARB	25 (39.7)	10 (47.6)	0.523
CCB	13 (20.6)	4 (19.1)	1.000
**Laboratories**			
Creatinine, µmol/L	78.60 (71.00, 88.00)	84.50 (69.80, 98.55)	0.383
eGFR, mL/min/1.73 m2	94.79 ± 15.46	86.68 ± 17.65	0.055
NT‐proBNP, pg/mL	47 (14, 122)	51 (25, 180)	0.800
**Indication for PCI**			
Stable angina	18 (28.6)	4 (19.0)	0.567
ACS–UA/NSTEMI	41 (65.1)	12 (57.1)	0.514
Previous MI	21 (33.3)	10 (47.6)	0.240
Previous PCI	28 (44.4)	9 (42.9)	0.899
TMIM frame count	22.45 ± 6.44	25.81 ± 6.71	0.044
CFVR	2.90 (2.60, 3.20)	2.30 (2.20, 2.40)	<0.001

Abbreviations: CFVR, coronary flow velocity reserve; PCI, percutaneous coronary intervention; BMI, body mass index; DAPT, dual antiplatelet therapy; ACE, angiotensin‐converting enzyme; ARB, angiotensin receptor blocker; CCB, calcium‐channel blocker; eGFR, estimated glomerular filtration rate; NT‐proBNP, N‐terminal pro‐B‐type natriuretic peptide; ACS‐UA/NSTEMI, acute coronary syndrome‐unstable angina/non‐ST‐segment‐elevation myocardial infarction; MI, myocardial infarction; TIMI, thrombolysis in myocardial infarction.

### 3.2 Echocardiographic Characteristics During Dipyridamole and Exercise

**FIGURE 2:**
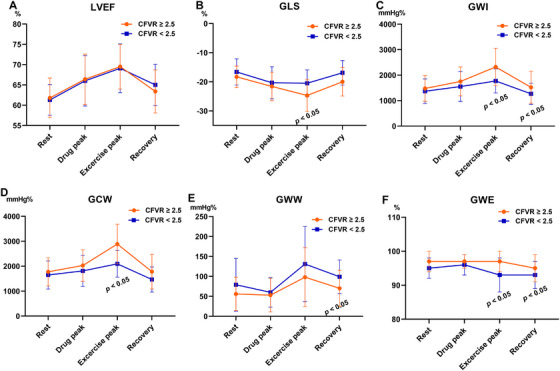
Serial changes of LVEF (A), GLS (B), GWI (C), GCW (D), GWW (E), and GWE (F) during dipyridamole‐exercise stress echocardiography in the two groups. CFVR, coronary flow velocity reserve; LVEF, left ventricular ejection fraction; GLS, global longitudinal strain; GWI, global work index; GCW, global constructive work; GWW, global wasted work, GWE, global work efficiency.

The changes in LV functional parameters across stress phases during DExE are summarized in Table 2, Figure 2, 3. Hemodynamic responses, including systolic blood pressure, diastolic blood pressure, and heart rate (HR), changed across stress stages in both groups (rest, drug peak, peak exercise, and recovery). However, the HR of the CFVR < 2.5 group in the recovery stage was higher than that of the CFVR ≥ 2.5 group (*p* = 0.015). No regional wall motion abnormalities were observed in either group during dipyridamole stress or peak exercise. However, the LVEF, GLS, GWI, GCW, GWW, and GWE values were significantly increased in the two groups. Specifically, the GLS, GWI, GCW, and GWE in the peak exercise stage of the CFVR < 2.5 group were lower than those in the CFVR ≥ 2.5 group (all *p* < 0.05). Moreover, during recovery, GLS, GWI, and GWE were lower, and GWW was higher in the CFVR < 2.5 group than in the CFVR ≥ 2.5 group (all *p* < 0.05).

**TABLE 2:** Comparison of LV function between the two groups during dipyridamole‐exercise stress echocardiography.

Parameters	Stress Phases	CFVR ≥ 2.5 Group (*n* = 63)	CFVR < 2.5 Group (*n* = 21)	*p‐*value
SBP, mmHg	Rest	116 ± 13	116 ± 12	0.953
	Drug peak	122 ± 14^a^	119 ± 16	0.504
	Exercise peak	157 ± 22^a^ ^,b^	148 ± 20^[Link]^, ^[Link]^	0.106
	Recovery	119 ± 16^c^	121 ± 13^c^	0.494
DBP, mmHg	Rest	76 ± 10	77 ± 10	0.623
	Drug peak	76 ± 10	73 ± 17	0.464
	Exercise peak	84 ± 11^[Link]^, ^[Link]^	80 ± 11	0.146
	Recovery	75 ± 11^c^	74 ± 13	0.859
HR, beats/minute	Rest	78 ± 14	83 ± 15	0.195
	Drug peak	90 ± 13^a^	95 ± 13^a^	0.144
	Exercise peak	117 ± 18^[Link]^, ^[Link]^	113 ± 19^[Link]^, ^[Link]^	0.351
	Recovery	90 ± 13^[Link]^, ^[Link]^	98 ± 14^[Link]^, ^[Link]^	0.015
LVEDVi, ml/m2	Rest	39.6 ± 9.4	38.4 ± 9.1	0.614
	Drug peak	37.9 ± 9.5	38.2 ± 7.8	0.885
	Exercise peak	35.6 ± 9.9^[Link]^, ^[Link]^	36.3 ± 10.0	0.781
	Recovery	36.7 ± 8.4^[Link]^, ^[Link]^	37.3 ± 9.5	0.795
LVEF, %	Rest	61.8 ± 4.9	61.3 ± 3.8	0.715
	Drug peak	66.4 ± 6.2^a^	66.0 ± 6.2^a^	0.785
	Exercise peak	69.5 ± 5.5^[Link]^, ^[Link]^	69.1 ± 6.0^[Link]^, ^[Link]^	0.772
	Recovery	63.4 ± 5.3^[Link]^, ^[Link]^, ^[Link]^	65.0 ± 5.1^[Link]^, ^[Link]^	0.244
E/e’	Rest	6.85 ± 2.21	7.26 ± 2.46	0.471
	Drug peak	7.25 ± 2.36	8.00 ± 2.34	0.208
	Exercise peak	7.52 ± 2.49	7.94 ± 2.38	0.497
	Recovery	6.60 ± 1.96^c^	7.64 ± 2.78	0.063
GLS, %	Rest	−18.3 ± 3.7	−16.6 ± 4.5	0.094
	Drug peak	−21.6 ± 4.9^a^	−20.3 ± 5.5^a^	0.322
	Exercise peak	−24.7 ± 5.5^[Link]^, ^[Link]^	−20.5 ± 4.6^a^	0.002
	Recovery	−20.0 ± 4.9^[Link]^, ^[Link]^, ^[Link]^	−16.9 ± 4.2^[Link]^, ^[Link]^	0.010
GWI, mmHg %	Rest	1479 ± 504	1369 ± 482	0.385
	Drug peak	1753 ± 567^a^	1552 ± 585^a^	0.165
	Exercise peak	2320 ± 734^[Link]^, ^[Link]^	1773 ± 472^a^	0.002
	Recovery	1523 ± 625^[Link]^, ^[Link]^	1268 ± 416^[Link]^, ^[Link]^	0.039
GCW, mmHg %	Rest	1775 ± 563	1647 ± 563	0.369
	Drug peak	2029 ± 634^a^	1812 ± 627	0.176
	Exercise peak	2887 ± 796^[Link]^, ^[Link]^	2095 ± 535^[Link]^, ^[Link]^	<0.001
	Recovery	1782 ± 693^[Link]^, ^[Link]^	1468 ± 505^[Link]^, ^[Link]^	0.060
GWW, mmHg %	Rest	52 (26, 71)	61 (37, 87)	0.141
	Drug peak	43 (29, 69)	55 (32, 87)	0.347
	Exercise peak	79 (44, 132)^[Link]^, ^[Link]^	108 (82, 142)^[Link]^, ^[Link]^	0.072
	Recovery	59 (34, 91)^c^	96 (70, 111)^[Link]^, ^[Link]^	0.003
GWE, %	Rest	97 (96, 99)	96 (93, 97)	0.053
	Drug peak	98 (96, 99)	97 (95, 98)	0.186
	Exercise peak	97 (95, 98)	95 (92, 97)^b^	0.003
	Recovery	97 (94, 98)^b^	93 (93, 96)^[Link]^, ^[Link]^	0.001
ΔLVEF (D − R), %		4.7 ± 3.8	4.7 ± 4.4	1.000
ΔLVEF (E − R), %		7.8 ± 4.6	7.8 ± 5.7	0.990
ΔGLS (D − R), %		−3.3 ± 2.5	−3.7 ± 3.2	0.545
ΔGLS (E − R), %		−6.5 ± 3.4	−3.9 ± 3.3	0.003
ΔGWI (D − R), mmHg%		274 ± 285	182 ± 281	0.203
ΔGWI (E − R), mmHg%		841 ± 469	404 ± 244	<0.001
ΔGCW (D − R), mmHg%		254 ± 336	165 ± 280	0.277
ΔGCW (E − R), mmHg%		1112 ± 485	448 ± 304	<0.001
ΔGWW (D − R), mmHg%		−3 ± 41	−20 ± 64	0.175
ΔGWW (E − R), mmHg%		41 ± 78	52 ± 91	0.596
ΔGWE (D − R), %		1 ± 3	1 ± 4	0.484
ΔGWE (E − R), %		0 ± 3	−2 ± 4	0.074

Abbreviations: SBP, systolic blood pressure; DBP, diastolic blood pressure; HR, heart rate; LVEDVi, left ventricular end‐diastolic volume index; LVESVi, left ventricular end‐systolic volume index; LVEF, left ventricular ejection fraction; GLS, global longitudinal strain; GWI, global work index; GCW, global contractive work; GWW, global waste work; GWE, global work efficiency; Δ(D − R), difference before vs. after dipyridamole stress; Δ(E − R), difference before vs. after exercise stress.

^a^ *p‐*value < 0.05 vs. Rest.

^b^ *p‐*value < 0.05 vs. Drug peak.

^c^ *p‐*value < 0.05 vs. Exercise peak.

^d^Main effect *p‐*value based on repeated measures of ANOVA for normal distributed variables.

**FIGURE 3:**
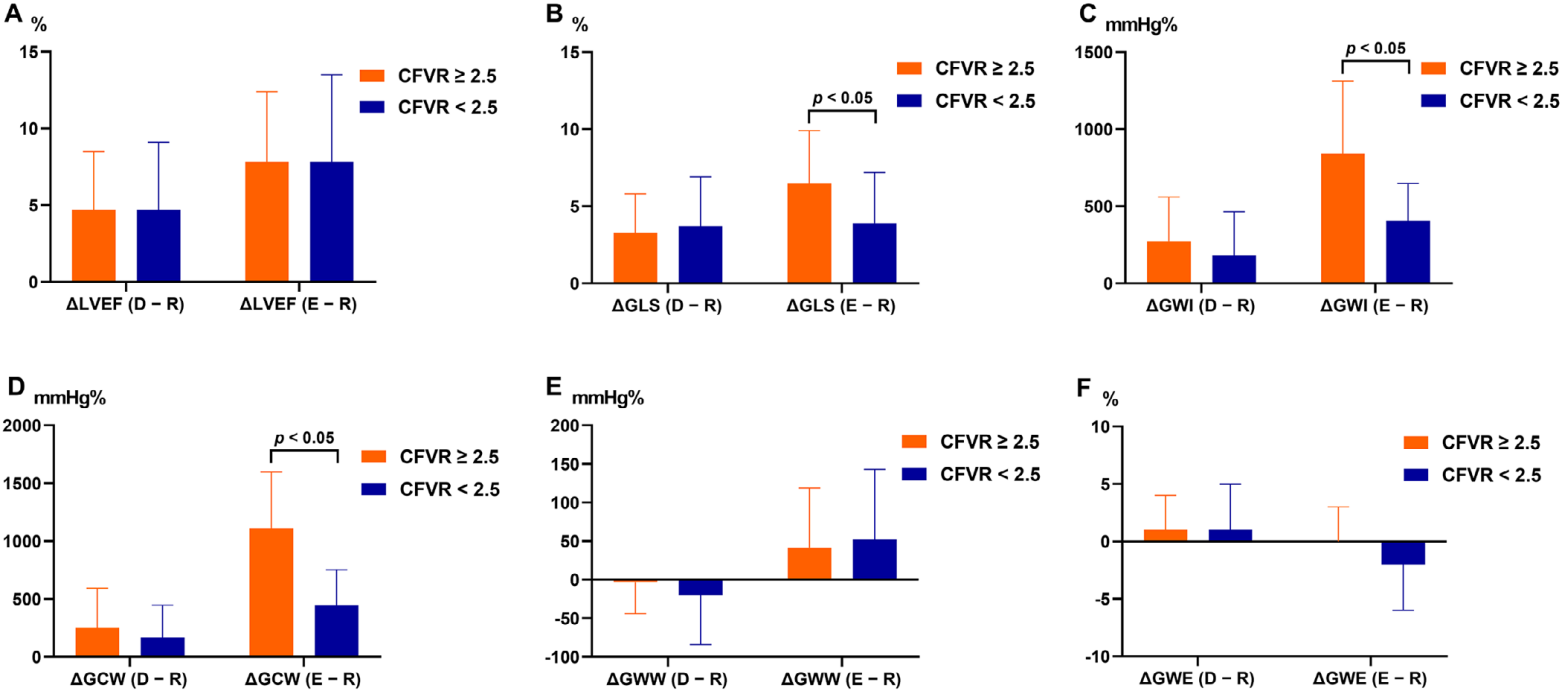
Difference in changes of cardiac function during dipyridamole‐exercise stress echocardiography in the two groups. CFVR, coronary flow velocity reserve; LVEF, left ventricular ejection fraction; GLS, global longitudinal strain; GWI, global work index; GCW, global constructive work; GWW, global wasted work, GWE, global work efficiency; Δ(D − R), difference before vs. after dipyridamole stress; Δ(E − R), difference before vs. after exercise stress.

**FIGURE 4:**
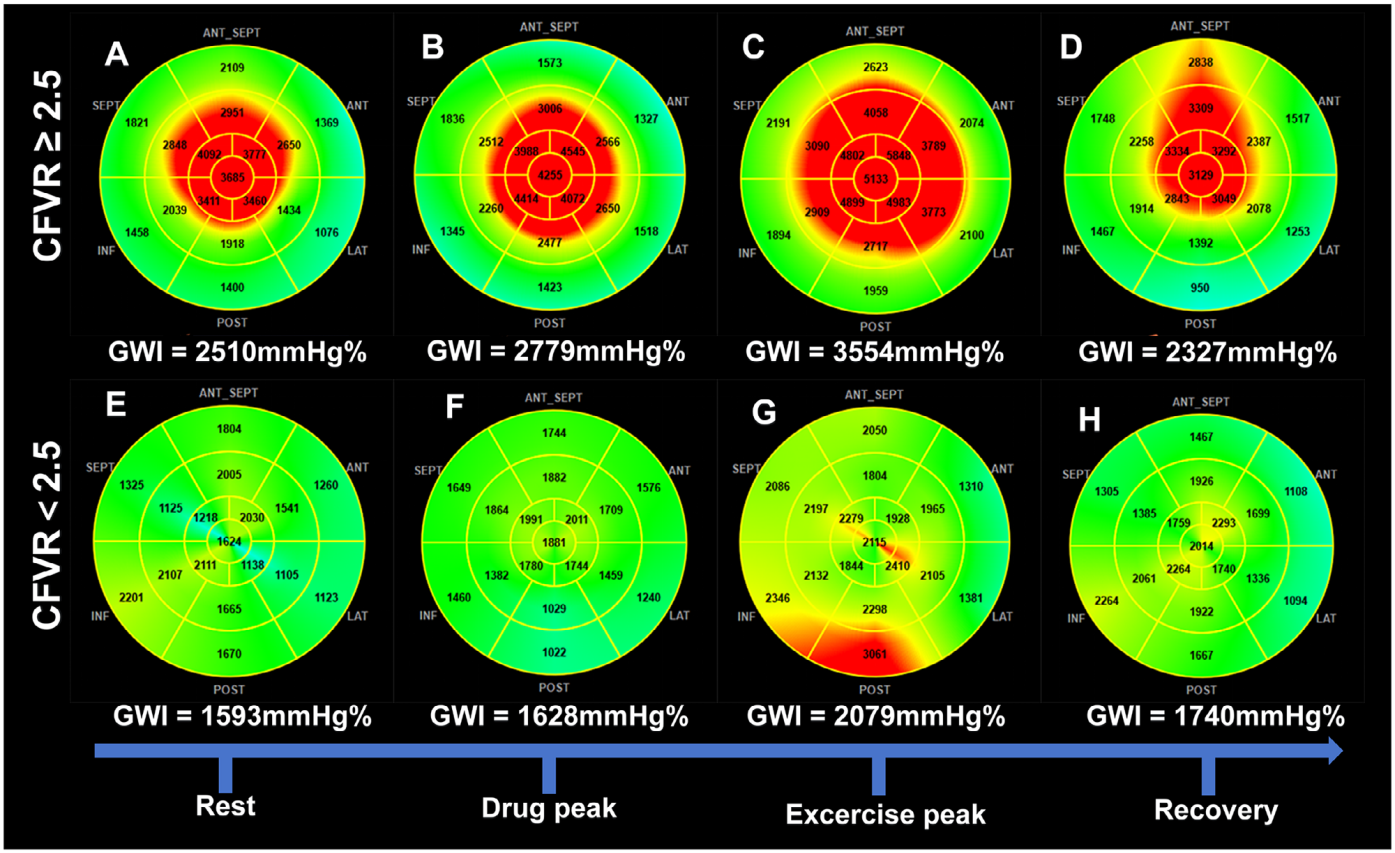
Changes in GWI during dipyridamole‐exercise stress: (A–D) in a 52‐year‐old female with CFVR ≥ 2.5; (E–H) in a 56‐year‐old female with CFVR < 2.5. CFVR, coronary flow velocity reserve; GWI, global work index.

Differences in LV functional reserve parameters between the two groups were observed following exercise‐induced stress (Figure 3). The absolute values of ΔGLS, ΔCWI, and ΔGCW in the CFVR < 2.5 group were significantly lower than those in the CFVR ≥ 2.5 group (all *p* < 0.05). Figure 4 and Figure S1 illustrate representative cases between the two groups, showing the changes in GLS and GWI during stress testing throughout the stress protocol.

Fifteen individuals (17.86%) experienced occasional atrial or ventricular premature beats during the stress process, which resolved after cessation of the stress test and rest. Thirty individuals (35.71%) presented with discomfort, primarily presenting as adverse drug reactions, such as chest tightness, palpitations, dizziness, fever, and nausea, which resolved after cessation of the stress test and rest or administration of aminophylline.

### 3.3 Independent Association of CFVR With Changes in Myocardial Strain/Work Indices During Stress

In Model 1, the TMIM frame count and multiple myocardial strain/work indicators at the peak exercise stage (GLS, GWI, GCW, and GWE), and some indicators at the recovery stage (GLS, GWW, and GWE) were significantly associated with CFVR < 2.5 (all *p* < 0.05) in univariable analyses. After multivariable adjustment, peak exercise GCW (OR = 0.99, 95% CI: 0.99–0.99, *p* = 0.008) and recovery GWW (OR = 1.05, 95% CI: 1.01–1.09, *p* = 0.027) remained independently associated with CFVR < 2.5.

**FIGURE 5:**
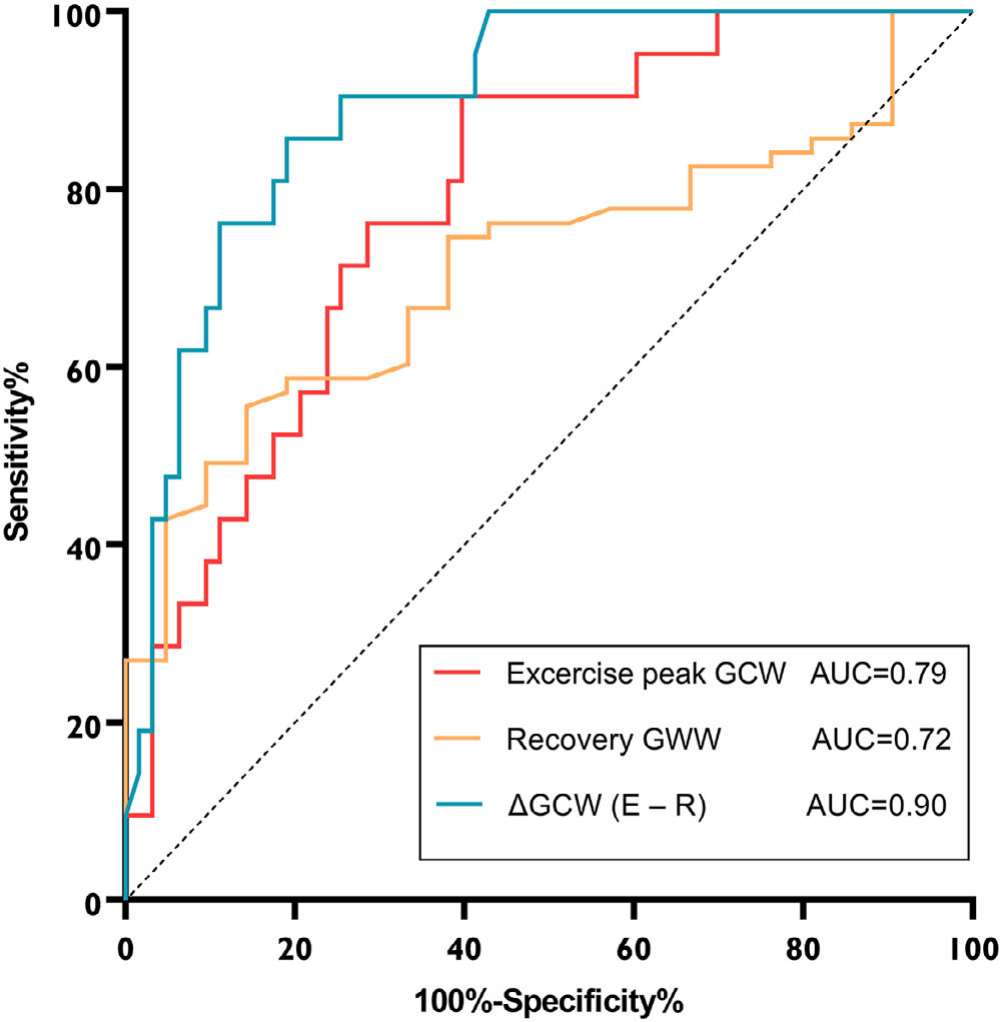
Receiver operating characteristic curves of myocardial work indices for detecting CFVR < 2.5. CFVR, coronary flow velocity reserve; GCW, global contractive work; GWW, global waste work.

In Model 2, the TMIM frame count, ΔGLS (E − R), ΔGWI (E − R), and ΔGCW (E − R) were associated with CFVR < 2.5 (all *p* < 0.05) in univariable analyses. After multivariable adjustment, ΔGCW (E − R) (OR = 0.99, 95% CI: 0.99–0.99, *p* = 0.003) was independently associated with CFVR < 2.5 (Table 3). According to the ROC analysis, ΔGCW (E − R) had the largest AUC (AUC: 0.90) in detecting CFVR < 2.5 (Figure 5).

**TABLE 3:** Logistic regression analysis showing the parameters associated with post‐PCI CFVR < 2.5.

Variables	Univariate analysis		Multivariate analysis	
	OR (95% CI)	*p*	OR (95% CI)	*p*
**Model 1: Excercise peak GCW + Recovery GWW**				
Age	1.06 (0.99 – 1.14)	0.103		
Sex	0.71 (0.18 – 2.80)	0.623		
Hypertension	2.00 (0.69 – 5.83)	0.204		
Dyslipidemia	0.75 (0.18 – 3.21)	0.698		
Diabetes	1.47 (0.50 – 4.28)	0.481		
ACS–UA/NSTEMI	0.72 (0.26 – 1.96)	0.515		
TMIM frame count	1.08 (1.01 – 1.17)	0.048	1.06 (0.94 – 1.18)	0.340
Excercise peak GLS, %	1.20 (1.06 – 1.37)	0.005	0.97 (0.72 – 1.32)	0.867
Excercise peak GWI, mmHg%	0.99 (0.99 – 0.99)	0.004	1.00 (1.00 – 1.01)	0.113
Excercise peak GCW, mmHg%	0.99 (0.99 – 0.99)	<0.001	0.99 (0.99 – 0.99)	0.008
Excercise peak GWE, %	0.78 (0.66 – 0.93)	0.005	1.12 (0.81 – 1.54)	0.490
Recovery GLS, %	1.17 (1.03 – 1.32)	0.015	1.22 (0.79 – 1.89)	0.367
Recovery GWI, mmHg%	1.00 (1.00 – 1.00)	0.090		
Recovery GWW, mmHg%	1.01 (1.01 – 1.02)	0.016	1.05 (1.01 – 1.09)	0.027
Recovery GWE, %	0.88 (0.79 – 0.99)	0.028	1.49 (0.91 – 2.43)	0.112
**Model 2: ΔGCW(E − R)**				
Age	1.06 (0.99 – 1.14)	0.103		
Sex	0.71 (0.18 – 2.80)	0.623		
Hypertension	2.00 (0.69 – 5.83)	0.204		
Dyslipidemia	0.75 (0.18 – 3.21)	0.698		
Diabetes	1.47 (0.50 – 4.28)	0.481		
ACS–UA/NSTEMI	0.72 (0.26 – 1.96)	0.515		
TMIM frame count	1.08 (1.01 – 1.17)	0.048	1.08 (0.97 – 1.21)	0.166
ΔGLS (E − R), %	1.27 (1.07 – 1.50)	0.006	0.92 (0.70 – 1.20)	0.521
ΔGWI (E − R), mmHg%	0.99 (0.99 – 0.99)	<0.001	1.00 (1.00 – 1.00)	0.736
ΔGCW (E − R), mmHg%	0.99 (0.99 – 0.99)	<0.001	0.99 (0.99 – 0.99)	0.003

Abbreviations: CFVR, coronary flow velocity reserve; PCI, percutaneous coronary intervention; ACS–UA/NSTEMI, acute coronary syndrome‐unstable angina/non‐ST‐segment‐elevation myocardial infarction; TIMI, thrombolysis in myocardial infarction; GLS, global longitudinal strain; GWI, global work index; GCW, global contractive work; GWW, global waste work; GWE, global work efficiency; Δ(D − R), difference before vs. after dipyridamole stress; Δ(E − R), difference before vs. after exercise stress.

### 3.4 Follow‐Up

**FIGURE 6:**
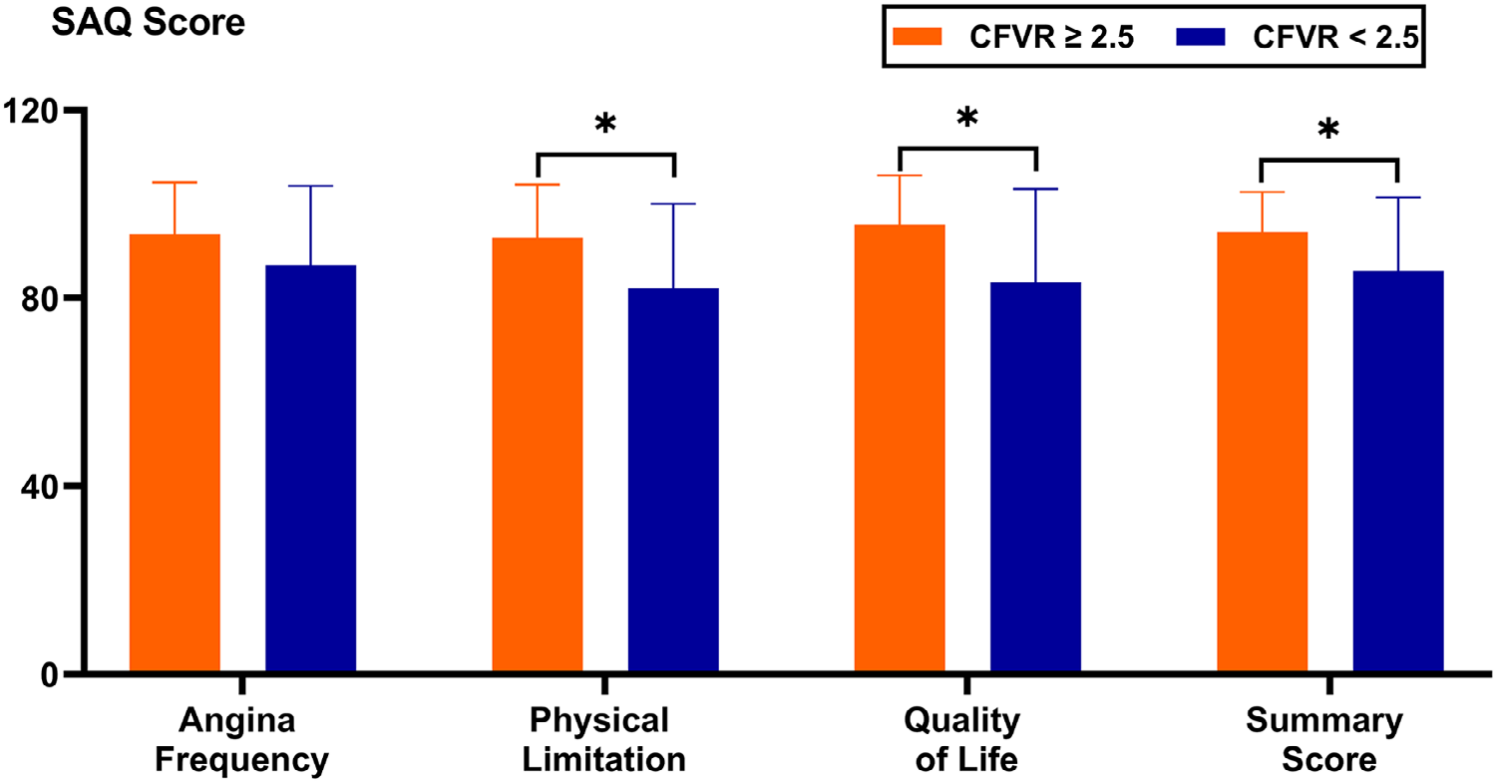
Early health status responses to PCI at 1 year in the two groups. CFVR, coronary flow velocity reserve; PCI, percutaneous coronary intervention; SAQ: Seattle Angina Questionnaire. *p < 0.05 vs. CFVR < 2.5.

No severe arrhythmias, heart failure, other adverse events or deaths occurred during the 1‐year follow‐up after PCI. Post‐PCI health status comparisons between the two groups are shown in Figure 6. At 1 year, the CFVR ≥ 2.5 group had higher SAQ physical limitation (92.86 ± 11.38 vs. 82.14 ± 17.93), quality of life (95.63 ± 10.57 vs. 83.33 ± 19.90), and summary score (94.05 ± 8.51 vs. 85.71 ± 15.71) than the CFVR < 2.5 group (all *p* < 0.05). No significant difference was observed for Angina Frequency (93.65 ± 10.97 vs. 86.90 ± 16.99, *p* = 0.124).

### 3.5 Intra‐ and Inter‐Observer Variability

Table S1 presents a comprehensive overview of the intra‐ and inter‐observer reliability of the GLS and MW indices. The substantial high ICCs reflect a strong level of agreement among observers within and across different assessments of the parameters.

## 4 Discussion

The main findings of this study are: (1) The combined DExE and myocardial strain/work protocol was feasible and safe. Approximately 25% of post‐PCI individuals with CAD with LVEF ≥ 50% experienced persistent CMD, which correlated with worse 1‐year physical limitation and quality of life. (2) Individuals in the CMD group demonstrated impaired contractility reserve, as evidenced by lower GLS, GWI, and GWE at peak exercise and during recovery; lower GCW at peak exercise; higher GWW during recovery; and smaller absolute changes Δ(E − R) in GLS, GWI, and GCW. Importantly, exercise peak GCW, recovery GWW, and ΔGCW (E − R) were independently associated with post‐PCI CMD, indicating underlying cardiac functional impairment in individuals with CMD. These findings highlight the need for closer cardiac function monitoring in these individuals.

In this study, we focused on individuals with a LVEF ≥ 50% after LAD‐PCI who achieved TIMI III and demonstrated that CMD persisted in 25% of them, as evidenced by significantly higher TIMI frame counts compared to non‐CMD individuals. Crucially, the individuals with CMD exhibited worse 1‐year clinical outcomes and impaired quality of life. Some 34.6% of individuals with ST‐segment elevation myocardial infarction (STEMI) exhibited persistent CMD, as quantified by an index of microcirculatory resistance (IMR) > 40, despite successful primary PCI [25]. Similarly, an IMR > 25 was present in 9.3% of individuals with non‐STEMI (NSTEMI) undergoing PCI and served as an independent predictor of major adverse cardiovascular events [26]. Pathological mechanisms, including endothelial dysfunction, microvascular spasm, inflammation, capillary drop off, and microcirculatory thrombosis drive CMD development. These mechanisms lead to inadequate myocardial perfusion, and ischemia and associated symptoms [27, 28]. Therefore, post‐PCI management should focus on epicardial vessel patency as well as assessment of microcirculatory function PCI.

The combined DExE protocol employed herein provides a safe and effective approach for the comprehensive evaluation of microcirculatory disturbance, myocardial ischemia, and cardiac contractile reserve. The integrated protocol utilized dipyridamole‐mediated hyperemia provided a validated approach for assessing CMD, whereas exercise stress induced myocardial ischemia and simultaneously enabled the quantification of cardiac functional reserve to distinguish between microvascular angina and epicardial ischemia. The utility of the DExE protocol has been suggested. Picano et al. [10] demonstrated that DExE protocol lowered the threshold for inducing myocardial ischemia and enhanced the detection rate of CAD compared to exercise stress testing alone. Moir et al. [11, 12] further explored DExE stress effectively identified impaired myocardial flow reserve caused by significant coronary artery stenosis. Piovesana et al. [13] provided evidence that DExE testing prolonged the development of relative ischemia to unmask significant ischemia in myocardial territories with borderline perfusion, improving prognostic value in CAD diagnosis. Importantly, no severe arrhythmias and uncontrollable complications occurred in our study. Symptoms such as chest tightness or palpitations were noted in several individuals and relieved after rest or aminophylline administration. The reversible and well‐tolerated nature of these effects further supports DExE's safety and clinical value.

In this study, the DExE protocol and 2D‐STE were used in tandem for multi‐parameter evaluation. We found no significant difference in the myocardial strain and work parameters of the two groups under the dipyridamole loading state. However, after combining dipyridamole and exercise stress, we observed differences in GLS, GWI, GCW, and GWE between the two groups. In our regression analysis, CMD was associated with exercise peak GCW and ΔGCW (E − R), suggesting that a single pharmacological stressor inducing the “coronary steal phenomenon” might be insufficient to provoke subtle cardiac functional alterations in individuals with CMD dysfunction. Putatively the combined exercise stress increased myocardial oxygen requirements beyond the microvascular reserve in CMD resulting in a perfusion deficiency that manifests as impaired LV contractile reserve and abnormal strain responses [29, 30]. Significant differences were observed in recovery GLS and GWE between the two groups, and the recovery GWW was independently associated with CMD in individuals after PCI. Abnormal cardiac responses may occur during the recovery phase [31, 32] emphasizing the importance of post‐test observation. One possible explanation is that individuals with CMD do not experience restoration of normal perfusion promptly, leading to persistent myocardial hypoperfusion [33]. Thus, the persistent compromise of myocardial contractile function by delayed hypoperfusion renders functional abnormalities during the recovery phase and is indicative of underlying CMD. These new and published data suggest that the DExE protocol comprehensively describes the physiological process from rest through peak stress to recovery, thereby significantly enhancing diagnostic sensitivity for detecting microvascular‐driven cardiac impairment.

Our study also focused on post‐PCI individuals with CAD with LVEF ≥ 50%. No abnormalities in segmental wall motion were observed during stress testing or throughout the stress protocol. However, impairments in contractile reserve were detected in individuals with CMD during the combined exercise stress (dipyridamole plus exercise) using myocardial strain and myocardial work indices. This is in line with the finding that individuals with CMD, despite having a lack of wall motion abnormalities under stress, show myocardial mechanical abnormalities specifically related to strain and work parameters [34, 35]. Notably, myocardial strain/work assessment provided a screen tool for clinically significant CAD and identified myocardial ischemia [32, 36, 37]. The enhanced sensitivity of myocardial strain and work indices enables the detection of latent microvascular dysfunction during stress testing, providing new imaging evidence for understanding the pathogenesis of persistent angina after PCI.

## 5 Limitations

This study has limitations. First, this is a single‐center study and the strict enrollment criteria focused solely on LAD lesions with a limited sample size. Verification of these findings by large‐scale multicenter studies would therefore be useful. Second, the measurement of myocardial strain and work parameters is affected by image quality. Thus, it is necessary to optimize the acquisition and analysis methods. Third, due to ethical reasons, exercise‐only was not tested. This precludes the definitive quantification of the incremental diagnostic value of DExE over isolated exercise testing for CMD‐related cardiac impairment. Nevertheless, DExE is a comprehensive modality for evaluating microcirculation‐driven cardiac dysfunction. Finally, the influence of CFVR on overall cardiac function was interrogated. Future studies should investigate the effect of CFVR on local 2D‐STE in the LAD artery blood supply area. Although DExE and 2D‐STE increase the potential cost, most individuals with CAD derived comparative benefits from this approach.

## 6 Conclusion

A combined DExE and myocardial strain/work evaluation protocol was found safe and informative among post‐PCI individuals with CAD with LVEF ≥ 50% with persistent CMD. The protocol improved the diagnostic sensitivity for microvascular‐driven cardiac impairment through complementary dipyridamole‐induced hyperemia and physiological exercise stress. Decreased CFVR was correlated with LV dysfunction, characterized by lower peak exercise GCW, higher recovery GWW, smaller ΔGCW (E − R), worse 1‐year physical limitation, and poorer quality of life. Future studies confirming this protocol's prognostic value should attention to close cardiac function monitoring in post‐PCI CMD individuals.

## Funding

This research was funded by the Yunnan Fundamental Research Kunming Medical University Projects (grant no. 202401AY070001‐165, grant no. 202401AY070001‐019), the Noncommunicable Chronic Diseases‐National Science and Technology Major Project (grant no. 2024ZD0527500) and the Yunnan Provincial Clinical Medicine Research Special Program (grant no. 202405AJ310003).

## Consent

Informed consent was obtained from all participants involved in the study.

## Conflicts of Interest

The authors declare no conflicts of interest.

## Ethics Statement

The study was conducted in accordance with the Declaration of Helsinki, and approved by the Institutional Ethics Committee of Yunnan Fuwai Cardiovascular Hospital (approval no. 2024‐013‐01; date of approval: March 29, 2024).

## Supporting information




**Figure S1**: Changes in GLS during dipyridamole‐exercise stress: (A–D) in a 52‐year‐old female with CFVR≥2.5; (E–H) in a 56‐year‐old female with CFVR<2.5;

Table S1: Inter‐ and intra‐observer variability of myocardial strain and work myocardial parameters.

## Data Availability

The data used in this study are available from the corresponding author.

## References

[1] J. S. Lawton , J. E. Tamis‐Holland , S. Bangalore , et al., “2021 ACC/AHA/SCAI Guideline for Coronary Artery Revascularization: A Report of the American College of Cardiology/American Heart Association Joint Committee on Clinical Practice Guidelines,” Circulation 145 (2022): e18–e114, 10.1161/CIR.0000000000001038. 34882435

[2] F. J. Neumann and M. Sousa‐Uva , “Ten Commandments' for the 2018 ESC/EACTS Guidelines on Myocardial Revascularization,” European Heart Journal 40 (2019): 79–80, 10.1093/eurheartj/ehy855. 30615155

[3] F. Crea , C. N. Bairey Merz , J. F. Beltrame , et al., “Mechanisms and Diagnostic Evaluation of Persistent or Recurrent Angina Following Percutaneous Coronary Revascularization,” European Heart Journal 40 (2019): 2455–2462, 10.1093/eurheartj/ehy857. 30608528

[4] C. A. Rajkumar , M. J. Foley , F. Ahmed‐Jushuf , et al., “A Placebo‐Controlled Trial of Percutaneous Coronary Intervention for Stable Angina,” New England Journal of Medicine 389 (2023): 2319–2330, 10.1056/nejmoa2310610. 38015442 PMC7615400

[5] R. Al‐Lamee , D. Thompson , H.‐M. Dehbi , et al., “Percutaneous Coronary Intervention in Stable Angina (ORBITA): A Double‐Blind, Randomized Controlled Trial,” Lancet 391, no. 10115 (2018): 31–40, 10.1016/S0140-6736(17)32714-9. 29103656

[6] G. Niccoli , R. A. Montone , G. A. Lanza , et al., “Angina After Percutaneous Coronary Intervention: The Need for Precision Medicine,” International Journal of Cardiology 248 (2017): 14–19, 10.1016/j.ijcard.2017.07.105. 28807510

[7] K. Wierzbowska‐Drabik , E. Picano , L. Cortigiani , et al., “Comparison of Coronary Flow Reserve Feasibility in Different Stress Echocardiography Protocols: Dobutamine, Dipyridamole, Exercise and Rapid Pacing,” Polish Archives of Internal Medicine 131, no. 9 (2021): 830‑839, 10.20452/pamw.16035. 34142788

[8] V. Kunadian , A. Chieffo , P. G. Camici , et al., “An EAPCI Expert Consensus Document on Ischaemia With Non‐Obstructive Coronary Arteries in Collaboration With European Society of Cardiology Working Group on Coronary Pathophysiology & Microcirculation Endorsed by Coronary Vasomotor Disorders International Study Group,” European Heart Journal 41, no. 37 (2020): 3504–3520, 10.1093/eurheartj/ehaa503. 32626906 PMC7577516

[9] D. M. Lopez , S. Divakaran , A. Gupta , et al., “Role of Exercise Treadmill Testing in the Assessment of Coronary Microvascular Disease,” JACC Cardiovascular Imaging 15, no. 2 (2022): 312–321, 10.1016/j.jcmg.2021.07.013. 34419395 PMC8831663

[10] E. Picano , F. Lattanzi , M. Masini , et al., “Usefulness of the Dipyridamole‐Exercise Echocardiography Test for Diagnosis of Coronary Artery Disease,” American Journal of Cardiology 62 (1988): 67–70, 10.1016/0002-9149(88)91366-5. 3381754

[11] S. Moir , B. A. Haluska , C. Jenkins , et al., “Incremental Benefit of Myocardial Contrast to Combined Dipyridamole‐Exercise Stress Echocardiography for the Assessment of Coronary Artery Disease,” Circulation 110, no. 9 (2004): 1108–1113, 10.1161/01.cir.0000139905.47128.9f. 15326066

[12] S. Moir , B. A. Haluska , C. Jenkins , et al., “Myocardial Blood Volume and Perfusion Reserve Responses to Combined Dipyridamole and Exercise Stress: A Quantitative Approach to Contrast Stress Echocardiography,” Journal of the American Society of Echocardiography 18, no. 11 (2005): 1187–1193, 10.1016/j.echo.2005.04.004. 16275528

[13] P. Piovesana , P. Offelli , F. D′Ambrosio , et al., “Addition of Exercise to Dipyridamole Stress Echocardiography in Order to Carry on the Ischemic Cascade: Role in the Diagnosis of Coronary Artery Disease and Prognostic Value,” Journal of Cardiovascular Echography 26, no. 4 (2016): 115–119, 10.4103/2211-4122.192173. 28465976 PMC5224665

[14] O. A. Smiseth , O. Rider , M. Cvijic , et al., “Myocardial Strain Imaging: Theory, Current Practice, and the Future,” JACC Cardiovascular Imaging 18, no. 3 (2025): 340–381, 10.1016/j.jcmg.2024.07.011. 39269417

[15] S. Roemer , A. Jaglan , D. Santos , et al., “The Utility of Myocardial Work in Clinical Practice,” Journal of the American Society of Echocardiography 34, no. 8 (2021): 807–818, 10.1016/j.echo.2021.04.013. 33895250

[16] C. Vrints , F. Andreotti , K. C. Koskinas , et al., “2024 ESC Guidelines for the Management of Chronic Coronary Syndromes,” European Heart Journal 45, no. 36 (2024): 3415–3537, 10.1093/eurheartj/ehae177. 39210710

[17] R. A. Byrne , X. Rossello , J. J. Coughlan , et al., “2023 ESC Guidelines for the Management of Acute Coronary Syndromes,” European Heart Journal 44, no. 38 (2023): 3720–3826, 10.1093/eurheartj/ehad191. 37622654

[18] P. Ong , P. G. Camici , J. F. Beltrame , et al., “International Standardization of Diagnostic Criteria for Microvascular Angina,” International Journal of Cardiology 250 (2018): 16–20, 10.1016/j.ijcard.2017.08.068. 29031990

[19] R. Sicari , P. Nihoyannopoulos , A. Evangelista , et al., “European Association of Echocardiography. Stress Echocardiography Expert Consensus Statement–Executive Summary: European Association of Echocardiography (EAE) (a registered branch of the ESC),” European Heart Journal 30 (2008): 278–289, 10.1093/eurheartj/ehn492. 19001473

[20] C. Mitchell , P. S. Rahko , L. A. Blauwet , et al., “Guidelines for Performing a Comprehensive Transthoracic Echocardiographic Examination in Adults: Recommendations From the American Society of Echocardiography,” Journal of the American Society of Echocardiography 32, no. 1 (2019): 1–64, 10.1016/j.echo.2018.06.004. 30282592

[21] J. U. Voigt , G. Pedrizzetti , P. Lysyansky , et al., “Definitions for a Common Standard for 2D Speckle Tracking Echocardiography: Consensus Document of the EACVI/ASE/Industry Task Force to Standardize Deformation Imaging,” European Heart Journal Cardiovascular Imaging 16, no. 1 (2015): 1–11, 10.1093/ehjci/jeu184. 25525063

[22] N. Marzlin , A. G. Hays , M. Peters , et al., “Myocardial Work in Echocardiography,” Circulation: Cardiovascular Imaging 16 (2023): e014419, 10.1161/circimaging.122.014419. 36734221

[23] H. M. Garcia‐Garcia , E. P. McFadden , A. Farb , et al., “Standardized End Point Definitions for Coronary Intervention Trials: The Academic Research Consortium‐2 Consensus Document,” Circulation 137 (2018): 2635–2650, 10.1161/circulationaha.117.029289. 29891620

[24] J. A. Spertus , J. A. Winder , T. A. Dewhurst , et al., “Development and Evaluation of the seattle angina Questionnaire: A New Functional Status Measure for Coronary Artery Disease,” Journal of the American College of Cardiology 25 (1995): 333–341, 10.1016/0735-1097(94)00397-9. 7829785

[25] M. El Farissi , F. M. Zimmermann , De Maria , et al., “The Index of Microcirculatory Resistance After Primary PCI: A Pooled Analysis of Individual Patient Data,” JACC Cardiovascular Interventions 16, no. 19 (2023): 2383–2392, 10.1016/j.jcin.2023.08.030. 37821183

[26] Y. Zhang , J. Pu , T. Niu , et al., “Prognostic Value of Coronary Angiography–Derived Index of Microcirculatory Resistance in Non–ST‐Segment Elevation Myocardial Infarction Patients,” JACC Cardiovascular Interventions 17, no. 16 (2024): 1874–1886, 10.1016/j.jcin.2024.04.048. 39115479

[27] V. R. Taqueti , “Coronary Flow Reserve: A Versatile Tool for Interrogating Pathophysiology, and a Reliable Marker of Cardiovascular Outcomes and Mortality,” European Heart Journal 43, no. 16 (2022): 1594–1596, 10.1093/eurheartj/ehac001. 35134171

[28] M. G. Del Buono , R. A. Montone , M. Camilli , et al., “Coronary Microvascular Dysfunction Across the Spectrum of Cardiovascular Diseases,” Journal of the American College of Cardiology 78, no. 13 (2021): 1352–1371, 10.1016/j.jacc.2021.07.042. 34556322 PMC8528638

[29] R. Senior and R. Khattar , “Stress Echocardiography: The Quest for Risk Stratification Beyond Myocardial Ischaemia,” European Heart Journal 42, no. 37 (2021): 3879–3881, 10.1093/eurheartj/ehab562. 34449836

[30] Q. Ciampi , A. Zagatina , L. Cortigiani , et al., “Prognostic Value of Stress Echocardiography Assessed by the ABCDE Protocol,” European Heart Journal 42, no. 37 (2021): 3869–3878, 10.1093/eurheartj/ehab493. 34449837 PMC8486488

[31] T. Takagi , A. Takagi , and J. Yoshikawa , “Detection of Coronary Artery Disease Using Delayed Strain Imaging at 5 Min After the Termination of Exercise Stress: Head to Head Comparison With Conventional Treadmill Stress Echocardiography,” Journal of Cardiology 55 (2010): 41–48, 10.1016/j.jjcc.2009.08.001. 20122547

[32] J. Lin , W. Wu , L. Gao , et al., “Global Myocardial Work Combined With Treadmill Exercise Stress to Detect Significant Coronary Artery Disease,” Journal of the American Society of Echocardiography 35, no. 3 (2022): 247–257, 10.1016/j.echo.2021.10.009. 34710569

[33] F. Mangiacapra , M. G. Del Buono , A. Abbate , et al., “Role of Endothelial Dysfunction in Determining Angina After Percutaneous Coronary Intervention: Learning From Pathophysiology to Optimize Treatment,” Progress in Cardiovascular Diseases 63, no. 3 (2020): 233–242, 10.1016/j.pcad.2020.02.009. 32061633

[34] H. Rodriguez‐Zanella , R. Arbucci , J. F. Fritche‐Salazar , et al., “Vasodilator Strain Stress Echocardiography in Suspected Coronary Microvascular Angina,” Journal of Clinical Medicine 11, no. 3 (2022): 711, 10.3390/jcm11030711. 35160163 PMC8836360

[35] Q. Liu , Q. Li , X. Wan , et al., “The Value of Myocardial Work in the Estimation of Left Ventricular Systolic Function in Patients With Coronary Microvascular Disease: A Study Based on Adenosine Stress Echocardiography,” Frontiers in Cardiovascular Medicine 10 (2023): 1119785, 10.3389/fcvm.2023.1119785. 37113699 PMC10126338

[36] A. Borrie , C. Goggin , S. Ershad , et al., “Noninvasive Myocardial Work Index: Characterizing the Normal and Ischemic Response to Exercise,” Journal of the American Society of Echocardiography 33 (2020): 1191–1200, 10.1016/j.echo.2020.05.003. 32651126

[37] A. Parlavecchio , G. Vetta , R. Caminiti , et al., “Which is the Best Myocardial Work Index for the Prediction of Coronary Artery Disease? A Data Meta‐Analysis,” Echocardiography 40, no. 3 (2023): 217–226, 10.1111/echo.15537. 36748264

