# Supplementary figures and images for: Impact of Coronary Microvascular Dysfunction on Left Ventricular Function After Percutaneous Coronary Intervention: Assessment With Combined Dipyridamole‐Exercise Stress and Myocardial Strain/Work
Source: Echocardiography. 2026 Mar 18;43(3):e70419. doi: 10.1111/echo.70419 (PMC12998498; doi:10.1111/echo.70419)

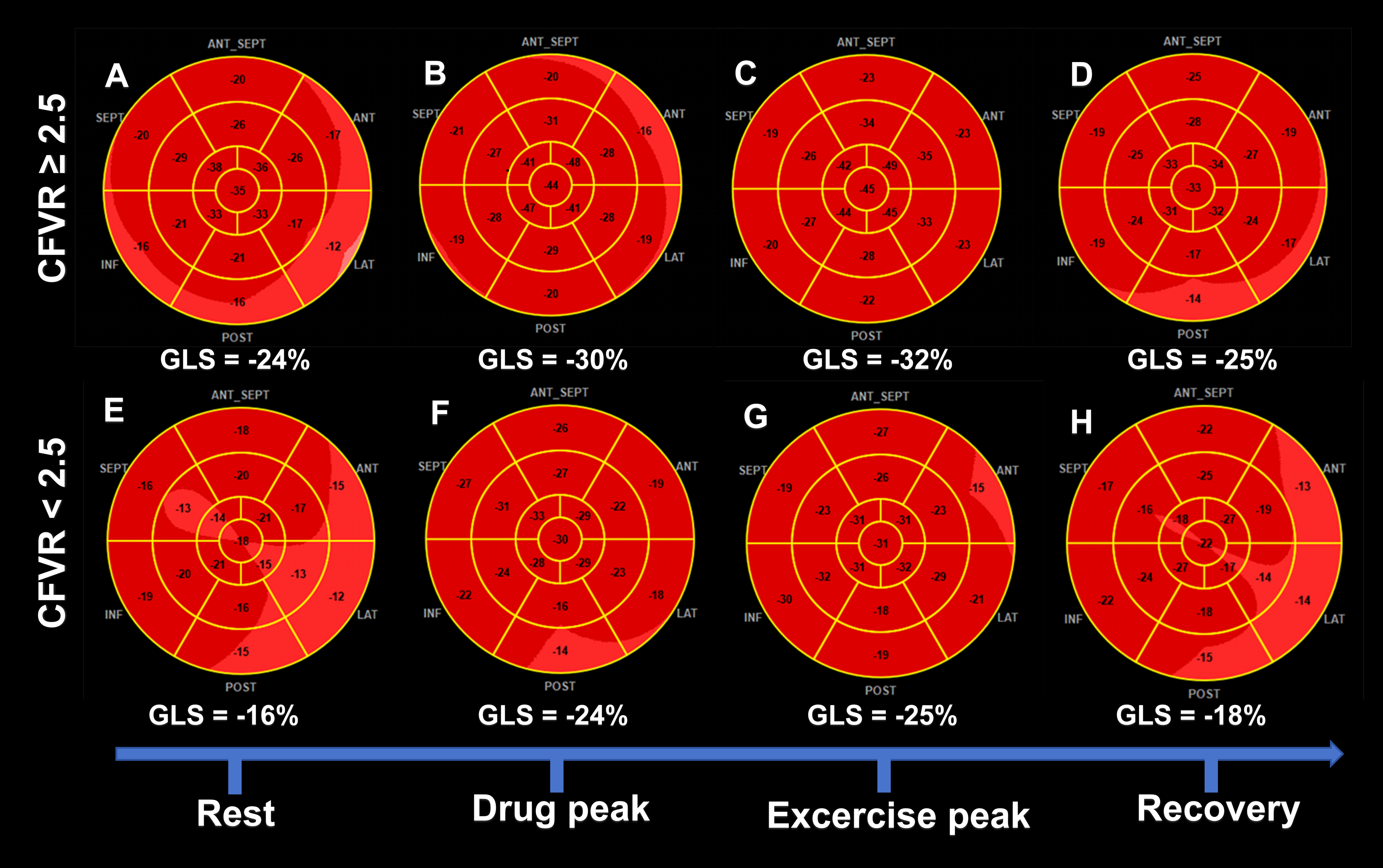

Supplement: Supplementary file 1 — Figure S1: Changes in GLS during dipyridamole‐exercise stress: (A–D) in a 52‐year‐old female with CFVR≥2.5; (E–H) in a 56‐year‐old female with CFVR<2.5; [file ECHO-43-e70419-s001.tif]
