# Supplementary material for: Impact of Coronary Microvascular Dysfunction on Left Ventricular Function After Percutaneous Coronary Intervention: Assessment With Combined Dipyridamole‐Exercise Stress and Myocardial Strain/Work
Source: Echocardiography. 2026 Mar 18;43(3):e70419. doi: 10.1111/echo.70419 (PMC12998498; doi:10.1111/echo.70419)
Supplement: Supplementary file 2 — Table S1: Inter‐ and intra‐observer variability of myocardial strain and work myocardial parameters. [file ECHO-43-e70419-s002.docx]

**Supplementary Table S1 Inter**- **and intra**-**observer variability of myocardial strain and work myocardial parameters**

| Parameters | Stress phases | Intra-class correlation coefficient | |
| --- | --- | --- | --- |
|  |  | Intra-observer | Inter-observer |
| GLS, % | Rest | 0.880 | 0.855 |
|  | Drug peak | 0.866 | 0.858 |
|  | Excercise peak | 0.823 | 0.858 |
|  | Recovery | 0.829 | 0.819 |
| GWI, mmHg% | Rest | 0.904 | 0.894 |
|  | Drug peak | 0.983 | 0.996 |
|  | Excercise peak | 0.958 | 0.972 |
|  | Recovery | 0.972 | 0.972 |
| GCW, mmHg% | Rest | 0.803 | 0.850 |
|  | Drug peak | 0.980 | 0.992 |
|  | Excercise peak | 0.891 | 0.901 |
|  | Recovery | 0.957 | 0.951 |
| GWW, mmHg% | Rest | 0.902 | 0.926 |
|  | Drug peak | 0.833 | 0.835 |
|  | Excercise peak | 0.968 | 0.971 |
|  | Recovery | 0.846 | 0.824 |
| GWE, % | Rest | 0.799 | 0.792 |
|  | Drug peak | 0.839 | 0.865 |
|  | Excercise peak | 0.816 | 0.808 |
|  | Recovery | 0.815 | 0.838 |

GLS, global longitudinal strain; GWI, global work index; GCW, global contractive work; GWW, global waste work; GWE, global work efficiency.
